# Editorial: Linking microbial-driven key processes with carbon and nitrogen cycling in estuarine, coastal, and the nearshore areas

**DOI:** 10.3389/fmicb.2024.1382148

**Published:** 2024-03-18

**Authors:** Genmei Lin, Dengzhou Gao, Ping Yang, Shuting Liu, Dongyao Sun, Xianbiao Lin

**Affiliations:** ^1^School of Marine Sciences, Sun Yat-sen University, Zhuhai, China; ^2^Key Laboratory for Humid Subtropical Eco-Geographical Processes of the Ministry of Education, School of Geographical Sciences, Fujian Normal University, Fuzhou, China; ^3^Department of Environmental and Sustainability Sciences, Kean University, Union, NJ, United States; ^4^School of Geography Science and Geomatics Engineering, Suzhou University of Science and Technology, Suzhou, China; ^5^Key Laboratory of Marine Chemistry Theory and Technology, Frontiers Science Center for Deep Ocean Multispheres and Earth System, Ministry of Education, Ocean University of China, Qingdao, China

**Keywords:** nitrogen cycle, carbon cycle, microbial community structure, functional diversity, marine ecosystems

The estuarine, coastal, and the nearshore areas are critical zones connecting terrestrial and marine ecosystems. Both natural processes and strong anthropogenic activities can influence material transformation, energy flow, as well as microbial and mineral interactions in these zones (Lazar et al., 2017; Cooke et al., 2020; Liu et al., 2020). Microbial community is one of the major drivers of biogeochemical cycles including carbon and nitrogen and plays an important role in ecological balance regulation in estuarine, coastal, and the nearshore ecosystems (Shiozaki et al., 2016; Sohm et al., 2016). Due to the close interrelationship between microorganisms and biogeochemical cycles, it is necessary to make deeper explorations about the coupling mechanism and the ecological effects in these environments. This interdisciplinary topic aims to understand the roles of microbial communities in processes such as organic matter decomposition, nutrient transformations, and greenhouse gas emissions (Lin and Lin, 2022; Zhang et al., 2023). By investigating the microbial drivers behind these key processes, we can gain insights into the functioning and resilience of estuarine, coastal, and the nearshore ecosystems and their responses to environmental changes. The seven articles in this Research Topic span a broad range of environments around the world, from the estuary and salt marshes to marine water and oxygen minimum zones, focusing on microbial community characteristics and the relevant carbon and nitrogen cycling processes.

This Research Topic includes studies on microbial taxonomic and functional profiles, which can provide fundamental understanding for microbial-driven biogeochemical processes. Niu et al. synthesized the information regarding the distribution patterns, assembly mechanisms, co-occurrence relationships, and ecological functions of bacterial communities from the Bohai Sea to the northern Yellow Sea. The key abiotic environmental factors shaping the communities included salinity, nutrient concentration (ammonium and nitrate), carbon content, total phosphorus, dissolved oxygen, and seawater turbidity. Meanwhile, the co-occurrence interactions among bacterial species also play vital roles in shaping bacterial community structures.

This Research Topic includes two publications on specific microbial-mediated carbon and nitrogen cycling processes analyzed using high-throughput sequencing strategy. The recycling of polymeric carbohydrates is a crucial process of carbon cycle in the ocean. Sun et al. investigated microbial CAZymes (carbohydrate-active enzymes) in Pearl River Estuary. The compositions of CAZymes gene were obviously distinct between water column and surface sediments, as well as between free-living bacteria and particle-associated bacteria in the water column. This indicated microbial glycan niche separation based on depth and size fraction, which could influence bacterial communities in coastal areas. Denitrification, the dominant process for nitrogen transformation mainly mediated by heterotrophic denitrifying bacteria, can remove more than half of annual nitrogen from the coasts. Li et al. focused on *nirK*- and *nirS*-type denitrifying bacterial communities in the situation of grazing prohibition in salt marshes. The results implied that grazing prohibition profoundly altered the diversity and abundance of *nirS*-type denitrifiers, whereas the counterparts of *nirK*-type groups remained relatively static. Therefore, instead of the *nirK* community, the *nirS* community could be a candidate microbial marker for grazing prohibition and salt marsh restoration.

Diazotroph-mediated N_2_ fixation could substantially relieve nitrogen limitation for primary production. Jiang et al. provided direct measurement of size-fractionated N_2_ fixation rates and identified controlling factors of these rates in the Changjiang (Yangtze River) Estuary. N_2_ fixation rates showed spatial variations and the bulk rates was correlated with ocean dynamic characteristics and the abundance of diazotrophs. High bulk rates were measured in the southeastern East China Sea where the intrusion of nitrogen-depleted Kuroshio water (Taiwan Warm Current and nearshore Kuroshio Branch Current) and abundant *Trichodesmium* were observed, while low rates occurred in the Changjiang Estuary where nitrogen-replete Changjiang Diluted Water and low abundance of *Trichodesmium* were observed. Raut et al. investigated benthic biological nitrogen fixation influenced by the decomposition of macroalgae and subsequent deposition on the seafloor. The measurements at the Big Fisherman's Cove on Santa Catalina Island, CA, USA implied the variations of unamended fixation rates among sites, which were linked to availability of organic matter as corroborated in bottle incubations. Through *nifH* sequencing, they showed that the diazotrophic community shifted with the decomposition and remineralization processes of macroalgae, from a community dominated by Gammaproteobacteria in the early period to a community dominated by Deltaproteobacteria, Bacteroidia, and Spirochaeta in the latter phase. The study clarified the effects of macroalgal loading and organic carbon on microbial communities especially coastal benthic diazotrophs.

The work of Parsons et al. used laboratory incubations to explore the characteristics of DOC (dissolved organic carbon) and prokaryotic community in response to the reoxygenation of suboxic waters. When the surface community was inoculated into water collected from the suboxic deep layer, this resulted in an increase of cell densities, the removal of DOC, and the shift of community and DOC quality. In comparison, the deep anaerobic inoculated community into surface water diminished by 25.5% initially, followed by an increase of cell density by 1.5-fold over 6 days. Also, a consistent shift of prokaryotic community and DOC quality occurred in both treatments. Therefore, this study proved the bioavailability of suboxic DOC to surface prokaryotes, which could influence biogeochemical cycles of biogenic elements including carbon and nitrogen.

DOM (dissolved organic matter) is essential in key processes of carbon and nitrogen cycles. Because of the high diversity in chemical compositions, Zhao focused on marine autochthonous FDOM (fluorescent DOM) and discussed its microbial origin. Microbes are the key source of allochthonous of marine FDOM. Furthermore, the author proposed future perspectives including harnessing multi-techniques for more precise and focused verifications of the chemical composition and structure of marine FDOM. Analyses of microbial cell structure and metabolism via genome and transcriptome strategies are also necessary.

The studies in this Research Topic covered multiple microbial-driven processes with carbon and nitrogen cycling via the combination of various approaches including biogeochemical measurements and multi-omics. Overall, the findings can expand the current understanding of microbial ecology as well as the biogeochemical cycle in estuarine, coastal, and the nearshore areas.

## Author contributions

GL: Writing – original draft, Writing – review & editing. DG: Writing – original draft, Writing – review & editing. PY: Writing – original draft, Writing – review & editing. SL: Writing – original draft, Writing – review & editing. DS: Writing – original draft, Writing – review & editing. XL: Writing – original draft, Writing – review & editing.
